# Using emergency department syndromic surveillance to investigate the impact of a national vaccination program: A retrospective observational study

**DOI:** 10.1371/journal.pone.0240021

**Published:** 2020-10-08

**Authors:** Helen E. Hughes, Alex J. Elliot, Thomas C. Hughes, Daniel Hungerford, Roger A. Morbey, Gillian E. Smith, Roberto Vivancos, Sarah J. O’Brien

**Affiliations:** 1 Real-time Syndromic Surveillance Team, Field Service, National Infection Service, Public Health England, Birmingham, United Kingdom; 2 Farr Institute at HeRC, University of Liverpool, Liverpool, United Kingdom; 3 John Radcliffe Hospital, Oxford, United Kingdom; 4 The Centre for Global Vaccine Research, Institute of Infection and Global Health, University of Liverpool, Liverpool, United Kingdom; 5 Field Epidemiology North West, Field Service, National Infection Service, Public Health England, Liverpool, United Kingdom; 6 School of Natural and Environmental Sciences, Newcastle University, Newcastle, United Kingdom; Ministry of Health and Sports, MYANMAR

## Abstract

**Background:**

Rotavirus infection is a common cause of gastroenteritis in children worldwide, with a high mortality burden in developing countries, particularly during the first two years of life. Rotavirus vaccination was introduced into the United Kingdom childhood vaccination schedule in July 2013, with high coverage (>90%) achieved by June 2016. We used an emergency department (ED) syndromic surveillance system to assess the impact of the rotavirus vaccination programme, specifically through the demonstration of any immediate and continuing impact on ED gastroenteritis visits in England.

**Methods:**

This retrospective, observational study used syndromic surveillance data collected from 3 EDs in the two years before (July 2011—June 2013) and 3 years post (July 2013—June 2016) introduction of rotavirus vaccination. The weekly levels of ED visits for gastroenteritis (by age group and in total) during the period before rotavirus vaccination was first described alongside the findings of laboratory surveillance of rotavirus during the same period. An interrupted time-series analysis was then performed to demonstrate the impact of rotavirus vaccination introduction on gastroenteritis ED visit levels.

**Results:**

During the two years before vaccine introduction ED visits for gastroenteritis in total and for the 0–4 years age group were seen to rise and fall in line with the seasonal rotavirus increases reported by laboratory surveillance. ED gastroenteritis visits by young children were lower in the three years following introduction of rotavirus vaccination (reduced from 8% of visits to 6% of visits). These attendance levels in young children (0-4years) remained higher than in older age groups, however the previously large seasonal increases in children were greatly reduced, from peaks of 16% to 3–10% of ED visits per week.

**Conclusions:**

ED syndromic surveillance demonstrated a reduction in gastroenteritis visits following rotavirus vaccine introduction. This work establishes ED syndromic surveillance as a platform for rapid impact assessment of future vaccine programmes.

## Introduction

Rotavirus infection is a common cause of gastroenteritis in children worldwide, particularly during the first two years of life. Clinical presentation ranges from mild, self-limited diarrhoea, to more serious cases requiring medical interventions, and deaths [1]. Although deaths are less likely in developed countries, illness due to rotavirus in the youngest children in the community results in high numbers of contacts with health care. In the United Kingdom (UK), rotavirus was estimated to account for much of the National Health Service (NHS) health care contacts made for acute gastroenteritis in children under 5 years: 27% of calls for advice (e.g. to the NHS 111 health advice line), 25% of visits to general practitioners (GPs), 20% of visits to emergency departments (ED) and 45% of hospital admissions [2]. Rotavirus is known to follow a seasonal pattern, with activity in the UK largely seen between January and June, usually reaching a peak in February/March (similar seasonal patterns are seen throughout Europe [3]).

Rotavirus vaccination (RV) with the live attenuated monovalent vaccine (Rotarix^®^: GlaxoSmithKline Biologicals) [4] was introduced into the UK childhood vaccination schedule in July 2013 [5] as a two dose course targeted at infants 8–15 weeks (second dose before 24 weeks) [6]. High coverage was achieved with >85% coverage for both doses by February 2014 [6], a level which increased to >90% by June 2016 [7]. Immediately following introduction of the RV programme reductions in the levels of gastroenteritis were reported in young children (0–4 years) in England, as estimated through laboratory confirmations, GP consultations and ED visits [8–12], with the costs avoided resulting in economic savings estimated at £12.5 million per year [13]. Similar results were reported in other countries including Australia [14], Brazil [15], Canada [16] and across sub-Saharan Africa [17] and Europe [18], although reductions were also reported in the Netherlands, where vaccination had not been introduced [19].

Syndromic surveillance involves the near real-time collection, analysis and reporting on health related data [20] which has been applied to a wide variety of contemporaneously collected patient data sources. This type of surveillance provides the potential to monitor and identify trends, across a wide variety of conditions and within shortened timescales compared to more traditional surveillance based on formal notifications and laboratory reporting. ED syndromic surveillance has previously demonstrated to be a valuable component in vaccine impact investigations alongside other data sources [9, 13, 21]. Here we demonstrate the utility of ED syndromic surveillance for a stand-alone investigation of a public health intervention: the introduction of rotavirus vaccination in England.

The principle aim of this study was to use a national ED syndromic surveillance system to assess the continued impact of the UK national RV programme. We first describe trends in ED visits for gastroenteritis during the two years prior to the introduction of the RV programme (2011–2013), compared to the weekly number of rotavirus confirmations identified in laboratory surveillance. We then explored the use of ED syndromic surveillance data for England to demonstrate the immediate impact of RV on young children attending EDs for gastroenteritis, to identify if previously reported reductions in rotavirus associated disease have continued. Our investigation also investigated possible changes in gastroenteritis ED visits across older age groups outside of the vaccination target groups, including any changes in seasonality.

## Methods

### Emergency department visits

The Emergency Department Syndromic Surveillance System (EDSSS) is part of the Public Health England (PHE) suite of real-time syndromic surveillance systems [22]. EDSSS was set up as a voluntary sentinel system prior to the 2012 London Olympic and Paralympic Games [23]. This system has provided an opportunity to investigate the ongoing impact of RV on ED visits, with surveillance data available from a number of English EDs, both prior to and following RV introduction.

The EDSSS collects an anonymised record for every visit at a participating ED on a daily basis, including: simple non-identifiable demographic data (sex and age), and any diagnoses selected. Clinical diagnoses are received in the coded format used within each ED; different diagnostic coding systems reveal different levels of clinical detail, requiring the development and use of a range of EDSSS syndromic indicators (three coding systems used in the sentinel EDSSS: NHS Accident and Emergency Diagnosis Tables [24], ICD10 [25] and Snomed-CT [26]). A detailed ‘gastroenteritis’ indicator (diagnosis codes considered to indicate an infectious gastrointestinal disease) was used here and was only available from those EDs reporting sufficiently detailed diagnostic codes (ICD10 or Snomed-CT: codes included in the gastroenteritis indicator, as reported by EDs included in this study, are detailed in S1 Table).

Only EDs able to report diagnosis codes mapped to the gastroenteritis indicator (gastrointestinal diagnoses considered due to infection), which reported throughout the time period and with no known changes in diagnosis coding practices or gaps in data, were eligible for inclusion.

The pre-RV period used for the description of gastroenteritis before RV programme introduction included data from July 2011 to June 2013. The post-RV period used for the investigation of vaccine impact included data from July 2013 to June 2016. Only EDs which were capable of reporting gastroenteritis throughout the pre-RV and post-RV time periods were eligible for inclusion in this study.

### Laboratory reports

Anonymised laboratory reports of rotavirus detection were accessed from the PHE Second Generation Surveillance System (SGSS), which contains data on isolates from diagnostic laboratories in England, using a range of diagnostic tests [27]. These data were used as an indicator of the community circulation of rotavirus during the two years prior to RV introduction available from EDSSS (4/7/11-30/6/13), ending the day before national RV implementation on 1/7/2013. Each laboratory report included the specimen date, patient age, organism identified and specimen type. Analyses were restricted to faecal specimens to exclude instances of invasive disease, which would not be comparable to the gastroenteritis ED visits. No restriction was included on specimen location (e.g. hospital/community) or patient age, as laboratory confirmation was used here to indicate pathogen activity in the community, not disease severity or age group affected. Episode based de-duplication is built into the SGSS [27], and therefore no further patient-based de-duplication was required.

### Descriptive analysis

Both ED syndromic surveillance and laboratory data for the two-year pre-RV period were grouped into weekly totals in order to remove any day of the week effects (04/7/11–03/07/16; International Organisation for Standardisation (ISO) weeks 2011 week 27 to 2013 week 26). The total weekly number of rotavirus isolates (as an indicator of community circulation) was compared to the weekly ED gastroenteritis visits in total and individual age group (0–4 years, 5–14 years, 15-44years, 45–64 years and 65+ years).

### Statistical analysis of vaccine impact on gastroenteritis ED visits

ED visit data, for number of visits with a gastroenteritis diagnosis and number of visits with a diagnosis code, were stratified by age group (as above) and by week. The number of total visits which included a diagnosis code each week was used as a denominator to calculate the percentage of visits due to gastroenteritis.

Time-series were constructed for the weekly percentage of visits reported as gastroenteritis for each age group and in total. An interrupted time-series analysis method was used to estimate the impact of the introduction of RV on gastroenteritis ED visits in each age group and for all ages. A negative binomial regression model, selected due to over dispersion, was fitted to the pre-vaccination period, to calculate estimated weekly visits, and an estimation of the trend and seasonality in the absence of vaccination, with the weekly gastroenteritis visits as the dependent variable. The total number of ED visits was included as an offset variable, to allow for changes in total ED visits over time and a seasonal harmonic (sine/cosine) Fourier pair of terms to model seasonality. These models were then projected forward to predict the expected visit levels had RV not been implemented. These ‘no change’, counterfactual models were then compared with models that included terms to account for a change following the vaccine introduction and a change in seasonality post-vaccine.

Interrupted time-series analysis was carried out using the statistical software R [28] (MASS, tsModel and epi packages [29–31]).

### Ethics

This surveillance is undertaken as part of the national surveillance functions of PHE and so ethical approval for this work was not required. The anonymised health data used in this study were routinely collected as part of the public health function of PHE.

## Results

Three EDs were eligible for inclusion in the study. They were based in two cities in England (one Northern, one Southern), included adult and paediatric services and reported consistently to EDSSS throughout both the pre-RV and post-RV periods. During the two years pre-RV, 596,122 visits (in the 3 study EDs) were reported to EDSSS, of which 71.5% included a diagnosis code (Table 1). In total, 2.2% of these coded visits were identified as due to gastroenteritis. The highest number of attendances for gastroenteritis were recorded in young children 0–4 years, despite this group accounting for only 10.2% of all ED visits (Table 1). Consequently, the percentage of attendances attributable to gastroenteritis was highest in children aged 0–4 years (8.0% of coded visits), whereas in age groups 5 years and over gastroenteritis was identified in less than 2.0% of ED visits (Table 1).

**Table 1:** ED visits, those including diagnosis coding and those identified as gastroenteritis, by age group during the pre-RV period from 4 July 2011 to 30 June 2013.

Age group	ED visits		Diagnosis included		Gastroenteritis visits	
	(% total visits)		(% age group visits)		(% age group visits with diagnosis)	
0–4 years	60,531	(10.2%)	43,354	(71.6%)	3,470	(8.0%)
5–14 years	49,623	(8.3%)	34,219	(69.0%)	655	(1.9%)
15–44 years	266,010	(44.6%)	189,907	(71.4%)	2,946	(1.6%)
45–64 years	104,615	(17.5%)	75,731	(72.4%)	909	(1.2%)
65+ years	114,672	(19.2%)	82,839	(72.2%)	1,431	(1.7%)
unknown	671	(0.1%)	308	(45.9%)	0	(0.0%)
Total	596,122	(100.0%)	426,358	(71.5%)	9,411	(2.2%)

**Fig 1:**
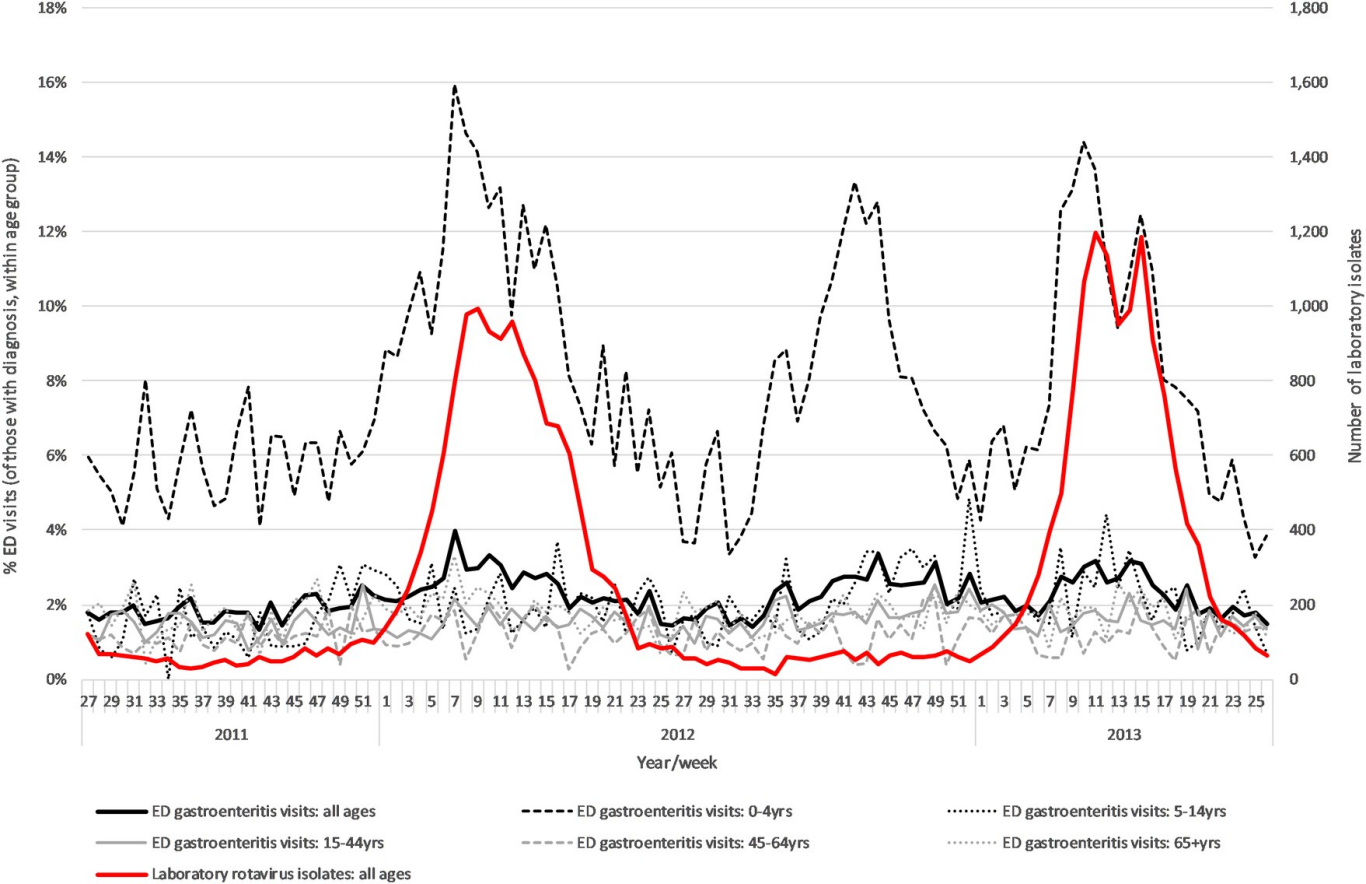
Weekly emergency department (ED) gastroenteritis visits (as a percentage of visits with a diagnosis), by age group and in total and weekly number of rotavirus laboratory isolations (England) during the two years pre-rotavirus vaccine introduction (2011 week 27–2013 week 26). Footnote: ED: emergency department; yrs: years.

A seasonal pattern was observed in gastroenteritis visits for children under 5 years during the 2-year pre-RV period, with increased ED attendances from week 1–17 each calendar year (Fig 1). This increase mirrored increases in rotavirus reported through laboratory surveillance during the same period. ED visits for gastroenteritis in all other age groups showed less seasonal variation (Fig 1).

A separate period of increased gastroenteritis visits was also observed during the summer of 2012 (week 39–45), particularly in children 0–4 years.

During the three years following the introduction of the rotavirus vaccine, 914,725 ED visits were reported by the three eligible EDs (Table 2). Diagnosis codes were received for 71.8% of visits (very similar to the levels identified during the pre-RV period), with 2.1% of these identified as due to gastroenteritis. The numbers and levels of gastroenteritis were highest in the youngest age group, 0–4 years though lower than identified during the pre-RV period (6.1% of visits with diagnosis information, compared to 8.0% before RV).

**Table 2:** Emergency department (ED) visits, those including diagnosis coding and those identified as gastroenteritis, by age group during the post-RV period from 1 July 2013 to 3 July 2016.

Age group	ED visits		Diagnosis included		Gastroenteritis visits		
	(% total visits)		(% age group visits)		(% visits with diagnosis)		
0–4 years	84,673	(9.3%)	63,411	(74.9%)	3,860	(6.1%)	
5–14 years	74,595	(8.2%)	52,385	(70.2%)	1,126	(2.1%)
15–44 years	401,187	(43.9%)	282,915	(70.5%)	4,863	(1.7%)
45–64 years	165,511	(18.1%)	119,562	(72.2%)	1,497	(1.3%)
65+ years	187,808	(20.5%)	137,815	(73.4%)	2,256	(1.6%)
unknown	951	(0.1%)	507	(53.3%)	0	(0.0%)
Total	914,725	(100.0%)	656,595	(71.8%)	13,602	(2.1%)

### Vaccine impact

**Fig 2:**
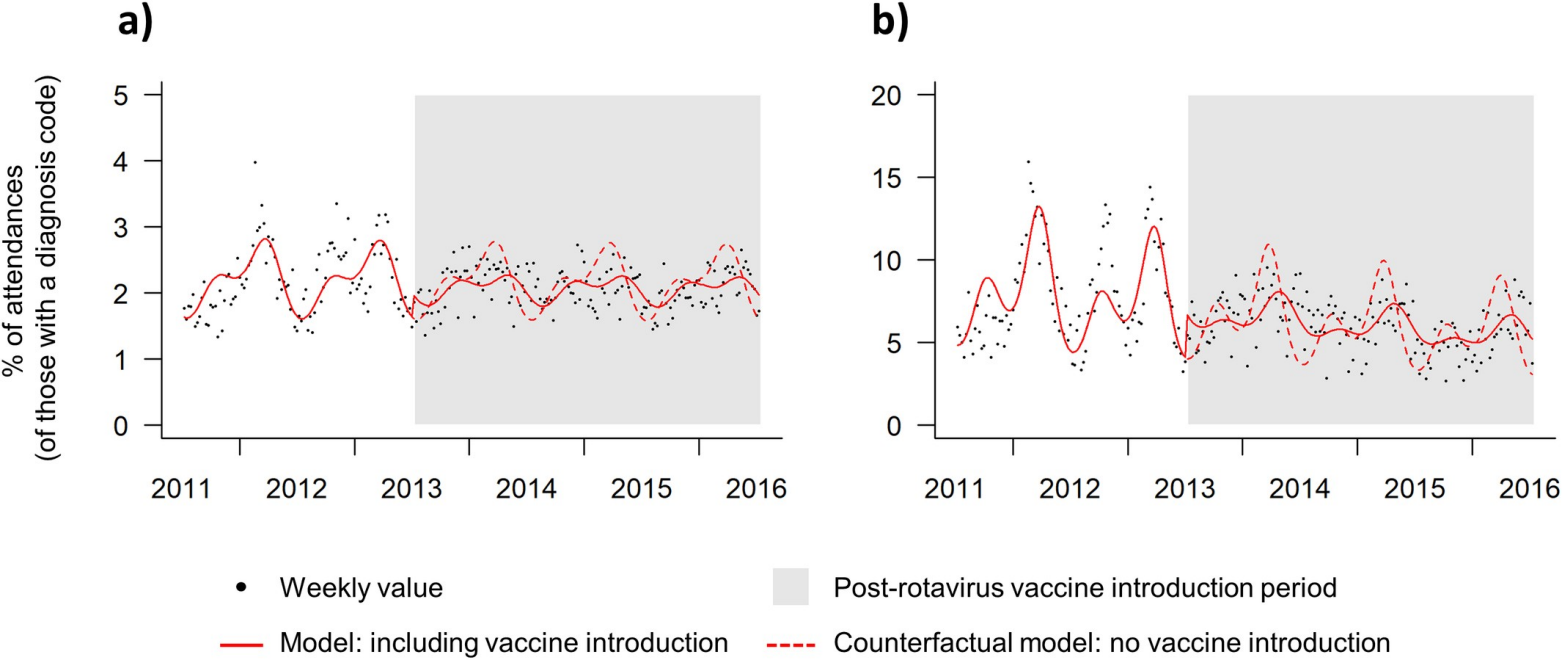
Weekly emergency department (ED) gastroenteritis visits, interrupted time-series regression model with level change and harmonic adjustment for seasonality, a) all ages and b) young children (0–4 years), week 27 2011 to week 26 2016 (grey box represents the rotavirus vaccine period).

The time-series constructed for gastroenteritis visits for all ages in total showed differences in both visit levels and seasonality between the pre-RV and post-RV time periods (Fig 2A). During the pre-RV period the weekly gastroenteritis levels ranged from 1.3–4.0% of all weekly visits. Post-RV slightly lower peaks were seen, ranging from 1.4–2.7% of all weekly visits (Fig 2A). As observed in the descriptive analysis, levels of gastroenteritis were much higher in young children (0–4 years; Fig 2B).

A more pronounced seasonal pattern was identified in ED visits present in young children (0–4 years: Fig 2B). The highest peaks in weekly visits levels were identified in this youngest age group (pre-RV max 15.9; post-RV max 9.6%: Fig 2B).

The interrupted time series models for all ages in total and for the 0-4years age group separately, demonstrated a clear divergence between the model fit to actual data and the counterfactual model (estimated trends had no vaccine been introduced). For the all age and 0–4 years group modelling the counterfactual models predicted large seasonal variation, as seen pre-RV. This degree of seasonality was not, however, seen in the post-RV period (Fig 2).

**Fig 3:**
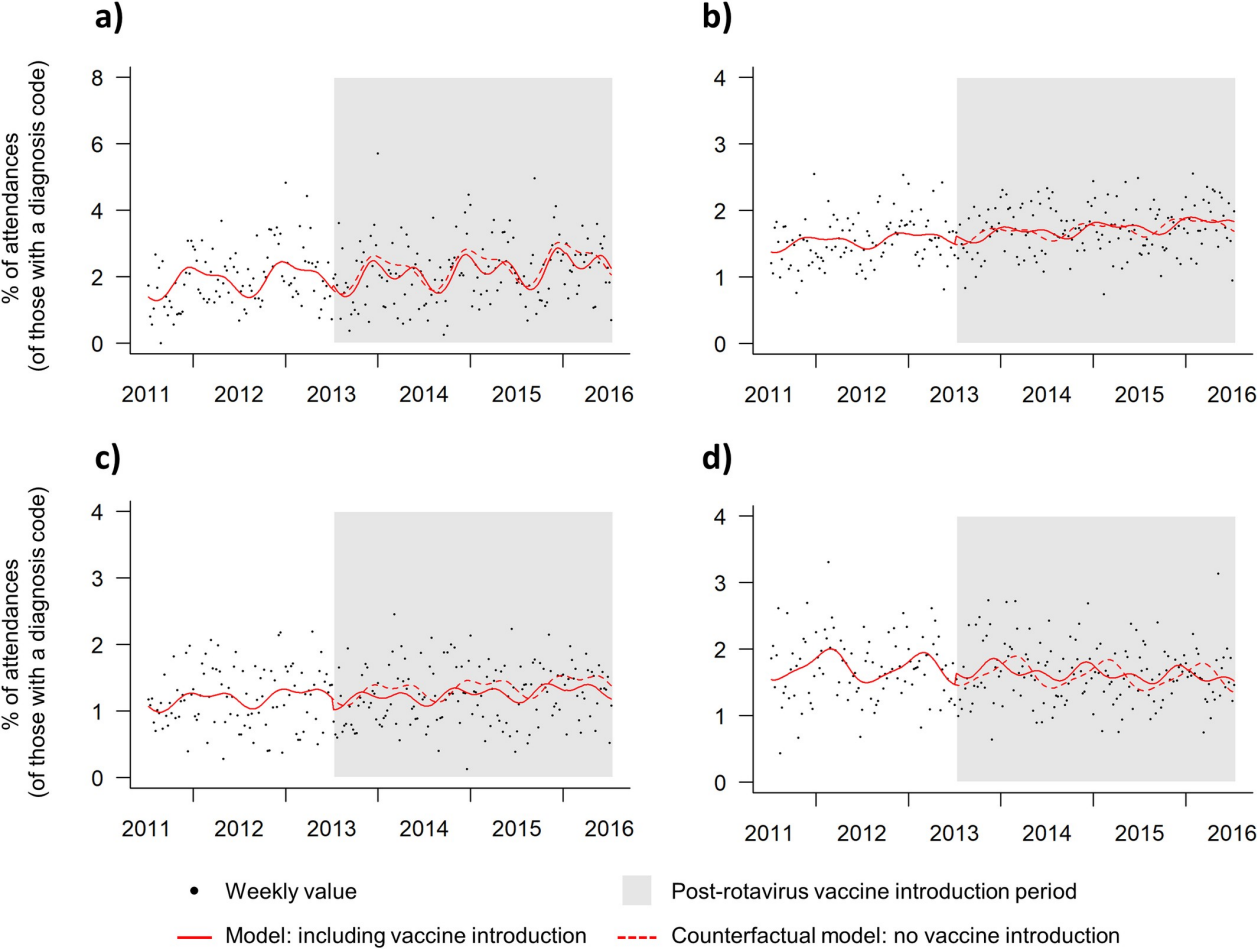
Weekly emergency department (ED) gastroenteritis visits, interrupted time-series regression model with level change and harmonic adjustment for seasonality, a) older children (5–14 years) and adults, b) 15–44 years, c) 45–64 years and d) 65+ years, week 27 2011 to week 26 2016 (grey box represents the rotavirus vaccine period).

In addition to the lowest levels of gastroenteritis visits being identified in other, older age groups (5+years) there was also less seasonal variation in visits, and less obvious differences between pre-RV and post-RV introduction. The modelling indicated similar results for the model fit to the actual data and the counterfactual model (Fig 3).

There was evidence of autocorrelation in the data, as would often be expected with time-series data. However, this was largely due to the seasonality observed in the data and removed by the introduction of a harmonic term into the models.

## Discussion

The descriptive time-series analysis of ED syndromic surveillance data identified seasonal trends in gastroenteritis ED visits in England prior to RV introduction, both for all ages in total and for young children. Gastroenteritis ED visits increased around the time of known seasonal rotavirus activity, as indicated by increased rotavirus laboratory confirmations. Prior to RV introduction, gastroenteritis levels in the youngest age group (0–4 years) were at much higher levels and showed greater seasonal variation than in older age groups. During periods of known rotavirus activity (2011 weeks 4–16, 2012 weeks 8–16) over 10% of ED visits (peaking at 16% of ED visits) made by children aged 0–4 years were identified as having a diagnosis of gastroenteritis.

The seasonal trends observed in all ages, and the high levels in young children implied a considerable burden of ED visits were associated with RV. This highlighted the usefulness of ED syndromic surveillance data for investigating the impact of rotavirus vaccine introduction into the childhood vaccination schedule.

Following the introduction of the national RV programme, the change in the seasonal variability of ED gastroenteritis visits was particularly notable in the youngest age group. The magnitude of the seasonal trend was reduced in comparison to the counterfactual model in the interrupted time series analysis, becoming more similar to the more stable (non-seasonal) trend observed in older age groups. Although gastroenteritis visits for young children (0–4 years) remained higher than older age groups, the variation week on week became attenuated, with smaller seasonal peaks (and troughs) observed in the ED data. This implies a change in the case mix of the youngest children seen in EDs, particularly during what had previously been recognised as the rotavirus season. This reduced level of gastroenteritis supports previous findings of a reduction in gastroenteritis immediately following RV introduction in both England [8–12] and other countries [14–18].

These results also highlight decreasing trends in ED attendances for gastroenteritis pre-vaccine, and post vaccine in the counterfactual model (i.e. in the absence of vaccine). Previous studies in England have demonstrated longer term falls in community-based general practitioner consultations for infectious intestinal disease [32, 33]. The findings here may indicate that public health messaging aimed at discouraging patients using health care services for mild self-limiting gastrointestinal infections, and changes in health care seeking behaviour is continuing to reduce the community burden from gastrointestinal infections on healthcare services.

There is evidence that introduction of rotavirus vaccination in infants may subsequently reduce gastroenteritis in adults [12, 18], however no clear decreases were observed in either the levels or seasonality of gastroenteritis visits in older age groups post-RV. The numbers of severely ill patients attending EDs may be too few to have a notable impact on ED workload. ED gastroenteritis visits levels for older children and adults continued to make up a smaller percentage of total visits in those age groups (0–6% for older children 5-14yrs, 0–3% for adults). The reduction in gastroenteritis attendances for young children did, however, result in reductions in the all age gastroenteritis attendances to EDs, changing the overall workload and case mix in EDs in general.

The observed reduction in ED gastroenteritis visits by young children reported here was not as great as the reductions reported in confirmed rotavirus hospitalisations [9, 13–18], though this was to be expected since ED syndromic surveillance gastroenteritis attendances are unlikely to be solely due to rotavirus. In the absence of a confirmatory testing (which is often unnecessary for successful treatment of gastroenteritis in an ED setting) there is no specific rotavirus syndromic indicator available; the gastroenteritis indicator used here for ED syndromic surveillance includes all pathogens and causes.

This work has further demonstrated the ability for non-pathogen specific syndromic surveillance to detect and describe a change in level of health seeking behaviour in the community for the more severe cases of illness (i.e. in the ED setting), following the introduction of a vaccine programme. During 2013 an initial pilot of the live attenuated influenza vaccine (LAIV) in the UK childhood vaccination programme used a range of different syndromic surveillance data (including ED attendances) to assess the impact and effectiveness [34]. The near real-time nature of ED syndromic surveillance data collection supported the timely assessment of LAIV impact in England, thereby supporting expansion of the pilot to the national immunisation programme.

### Strength and weaknesses

The EDSSS provides the potential to identify, quantify and monitor the levels of illness in the population requiring ED care. As the largest proportion of those affected by rotavirus infection do not need ED care i.e. they ‘self-treat’ [2], the numbers of cases eligible for inclusion in this study were limited and the findings should not be extended to estimate levels of less severe illness in the community. Despite the non-specific nature of syndromic surveillance, reliant on a preliminary/low detail/non-specific diagnoses form EDs (eg ‘gastroenteritis’ rather than confirmed rotavirus infection), clear trends in presentations of illness were identified here that coincided with rotavirus seasonality.

We have shown here the utility of EDSSS in monitoring the likely impact of rotavirus activity, despite the system itself being limited by the data available at both geographical coverage/number of EDs and the time periods available. The EDSSS was established to support the 2012 London Olympic and Paralympic Games using routinely collected data in a standardised format, allowing for identification of gastroenteritis in geographically distinct locations. Changes in system coverage and local work practices were unavoidable. Though individual EDs did provide data from late July 2010 both the pre-RV and post-RV data had to be limited to include data reported to EDSSS from only those EDs reporting consistently. This resulted in the inclusion of data from 3 EDs which reported from 2011 week 27 to 2016 week 26 in this study.

Syndromic surveillance in general is limited by the availability and quality of the data received. Here we included young children in the analysis as a 0–4 years age group. In the year following introduction of vaccine, the 0–4 years age group used here would have included those infants in the vaccine cohort and those who would not have received vaccine. Refining the analysis by year of age may have illustrated an increased impact of RV, however it was not possible to use a finer resolution of age (by year) in this youngest group using the data received in EDSSS for the time periods under investigation. Furthermore, with near real-time data extraction there is potential for incomplete records where the patient is still on their journey through the ED, so there may be no recorded diagnosis at the time of data extraction. The reasons for these gaps are unknown. Although the causes may be ED specific, it is assumed that they are also a constant in each site, allowing for comparison on trends over time. No changes in diagnosis data quality were identified in the EDs included in the analysis reported here.

As ED records do not routinely include information on vaccine status it was not possible to ascertain the vaccine status of those ED patients during the study period.

### Future work

ED syndromic surveillance systems exist in a number of different countries. Previous collaborative work has shown these systems to be compatible, with syndromic indicators used to describe and compare trends across international borders [35, 36], giving opportunity for similar work on the impact of vaccination implementation on ED visits on a larger scale.

A second period of increased gastroenteritis visits was identified during the pre-RV time period, particularly in those aged 0–4 years during September-October 2012. These increases may indicate increased activity of other gastrointestinal pathogens and coincided with increased seasonal laboratory reporting of cryptosporidium [37]. This suggests that ED surveillance may be of use in identifying periods of increased gastrointestinal pathogen activity in the community, which merits further exploration.

The introduction of the Emergency Care Data Set in England during 2018 has provided further opportunities for EDSSS [38]. The newly standardised, routine, mandated collection of emergency care data has widened the potential of EDSSS as a surveillance tool by creating a data source capable of providing the data required for long term studies of public health importance. By January 2020, the sentinel EDSSS described here had developed from a voluntary, sentinel surveillance system with limited coverage, to the national EDSSS; with almost every ED in England providing data. This development opens the possibility for using ED syndromic surveillance in future rapid studies on the impact of public health interventions. Such examples include the future introduction of a respiratory syncytial virus (RSV) vaccine: EDSSS has previously been shown to be sensitive to increases in RSV circulation in the community thus making it a suitable tool for monitoring impact post-vaccine implementation [39]. Additionally, EDSSS has recently been used to monitor the impact of interventions used during COVID-19 pandemic in England. Social distancing and shielding measures alongside changes in guidance on how the public accessed health care services were introduced in England during March 2020. EDSSS was able to provide real-time intelligence on the impact of these restrictions, demonstrating significant decreases in patient attendances in EDs in England during the period of the COVID-19 intervention [40–42].

## Supporting information

S1 Table Diagnostic codes mapped to the gastroenteritis syndromic surveillance indicator included in the EDSSS and used in the study. (DOCX)
