# Supplementary material for: Using emergency department syndromic surveillance to investigate the impact of a national vaccination program: A retrospective observational study
Source: PLoS One. 2020 Oct 8;15(10):e0240021. doi: 10.1371/journal.pone.0240021 (PMC7544051; doi:10.1371/journal.pone.0240021)
Supplement: S1 Table — (DOCX) [file pone.0240021.s001.docx]

**Supplementary Table 1:** Diagnostic codes mapped to the gastroenteritis syndromic surveillance indicator included in the EDSSS and used in the study.

| **Codes*** | **Codesystem** |
| --- | --- |
| A09, R11.X | **ICD-10^1^** |
| 62315008, 249519007, 75258004, 25374005, 111407006, 266071000, 16932000, 83227006, 11840006, 111843007, 422400008 | **SnomedCT^2^** |

*Only codes actually reported to EDSSS and used in this analysis are shown, additional relevant codes may exist

**References**

1. World Health Organisation. International Classification of Disease (ICD) 2010 [Available from: <http://www.who.int/classifications/icd/en/>.

2. International Health Terminology Standards Development Organisation. SNOMED CT 2012 [Available from: <http://www.ihtsdo.org/snomed-ct/>.
